# Competing Endogenous RNA (ceRNA) Networks and Splicing Switches in Cervical Cancer: HPV Oncogenesis, Clinical Significance and Therapeutic Opportunities

**DOI:** 10.3390/microorganisms10091852

**Published:** 2022-09-16

**Authors:** Afra Basera, Rodney Hull, Demetra Demetriou, David Owen Bates, Andreas Martin Kaufmann, Zodwa Dlamini, Rahaba Marima

**Affiliations:** 1 SAMRC Precision Oncology Research Unit (PORU), DSI/NRF SARChI Chair in Precision Oncology and Cancer Prevention, Pan African Cancer Research Institute (PACRI), University of Pretoria, Hatfield, Pretoria 0028, South Africa; 2 Department of Medical Oncology, Steve Biko Academic Hospital and University of Pretoria, Hatfield, Pretoria 0028, South Africa; 3 David Owen Bates, Division of Cancer and Stem Cells, Centre for Cancer Sciences, Biodiscovery Institute, University of Nottingham, Nottingham NG7 2RD, UK; 4 Clinic for Gynaecology, Laboratory for Gynaecologic Tumor Immunology, Institute of Health, Charité-Universitätsmedizin, Freie Universität Berlin, Humboldt-Universität zu Berlin, Augustenburgerplatz 1, 13353 Berlin, Germany

**Keywords:** human papillomavirus (HPV), cervical cancer (CC), competing endogenous RNAs (ceRNAs), alternative splicing (AS), immunosuppression, low middle-income countries (LMICs), protein arginine methyltransferases (PMRTs), splicing disruptor drugs

## Abstract

Cervical cancer (CC) is the primary cause of female cancer fatalities in low-middle-income countries (LMICs). Persistent infections from the human papillomavirus (HPV) can result in cervical cancer. However, numerous different factors influence the development and progression of cervical cancer. Transcriptomic knowledge of the mechanisms with which HPV causes cervical cancer pathogenesis is growing. Nonetheless, there is an existing gap hindering the development of therapeutic approaches and the improvement of patient outcomes. Alternative splicing allows for the production of numerous RNA transcripts and protein isoforms from a single gene, increasing the transcriptome and protein diversity in eukaryotes. Cancer cells exhibit astounding transcriptome modifications by expressing cancer-specific splicing isoforms. High-risk HPV uses cellular alternative splicing events to produce viral and host splice variants and proteins that drive cancer progression or contribute to distinct cancer hallmarks. Understanding how viruses utilize alternative splicing to drive pathogenesis and tumorigenesis is essential. Although research into the role of miRNAs in tumorigenesis is advancing, the function of other non-coding RNAs, including lncRNA and circRNA, has been understudied. Through their interaction with mRNA, non-coding RNAs form a network of competing endogenous RNAs (ceRNAs), which regulate gene expression and promote cervical cancer development and advancement. The dysregulated expression of non-coding RNAs is an understudied and tangled process that promotes cervical cancer development. This review will present the role of aberrant alternative splicing and immunosuppression events in HPV-mediated cervical tumorigenesis, and ceRNA network regulation in cervical cancer pathogenesis will also be discussed. Furthermore, the therapeutic potential of splicing disruptor drugs in cervical cancer will be deliberated.

## 1. Introduction

**Figure 1:**
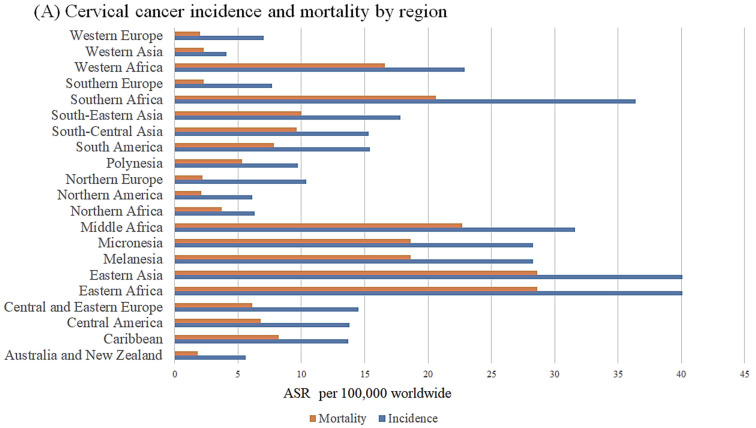

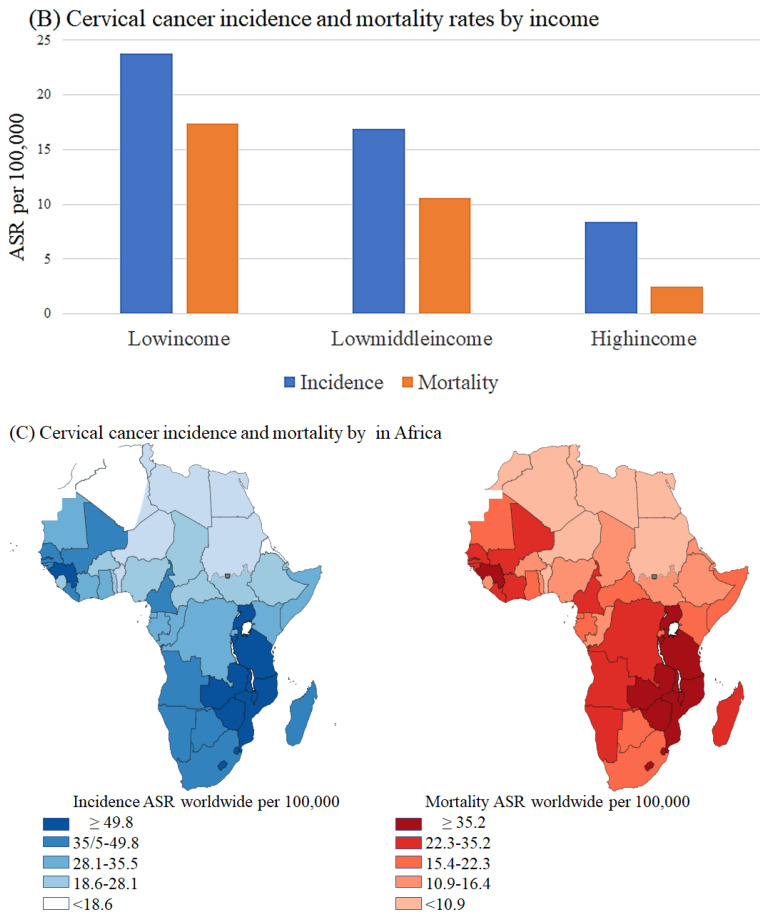
**Cervical epidemiology- HICs vs. LMICs**. (**A**) Incidence and mortality rates of cervical cancer per region. (**B**) Incidence and mortality rates based on income (**C**) Map depicting the age-standardized rate (ASR) for incidence and mortality per individual country in Africa, representing LMICs [6].

Cervical cancer was the fourth most commonly diagnosed cancer and the fourth leading cause of cancer fatalities in women in 2020, with incidence and mortality rates of 3.1% and 3.4%, respectively. In 2020, an incidence of 604,000 and mortality of over 342,000 was reported globally [1]. Disproportionality in mortality and incidence between countries with high income and countries with low and middle income has earned the disease the name "disease of disparity." Low-middle-income countries (LMICs) carry the brunt of the disease, contributing to approximately 85% of the new cases [2,3] (Figure 1). In Sub-Saharan Africa (SSA), cervical cancer is the second-most common cause of cancer-related death in women. [4]. Southern Africa has one of the highest age-standardized incidence rates (ASR) of CC in the world (43.1 per 100,000) [5].

To fully comprehend the disparity of cervical cancer in LMICs, one must focus on the numerous challenges in implementing cervical cancer management strategies. Governments in LMICs are faced with the co-prioritization of health care with other priorities, including education, water, electricity, and infrastructure development [2,7]. Infrastructure to prevent disease progression and advanced management may not exist nationally in LMICs such as SSA. In the presence of infrastructure, other cultural factors, including stigmatization, and Indigenous and religious beliefs, may hinder the sick from accessing the necessary resources [2,8].

Several risk factors are implicated in cervical cancer. These include smoking, multiple sexual partners, Human Immune deficiency Virus (HIV), and prolonged use of oral contraceptives [8,9]. However, HPV is the leading identified cause of cervical cancer tumorigenesis [10]. HPV infections are common in sexually active females between 18 and 30 [11]. Persistent infections from high-risk HPV are etiologically responsible for invasive cervical, oropharyngeal [10,12], vulva [13], penile [14,15], head and neck, anal and vulvovaginal cancers [16]. Clinical interventions used to treat cervical cancer depend on cancer’s clinical stage. Early-stage cervical cancer is treated primarily by surgery and radiation therapy. Surgery is reserved for early-stage malignancy, fertility retention, and smaller lesions like stage IA, IB1, IB2, and identified IIA1 [17,18].

Stages IB3, II, III, and IVA malignancy are treated with concurrent chemoradiation with platinum-containing chemotherapy (cisplatin alone or cisplatin/fluorouracil) [17,19]. For more advanced malignancies, radiotherapy and systemic treatment are considered [19,20]. Distant metastatic disease (i.e., stage IVB) is frequently treated with platinum-containing chemotherapy. In contrast, patients without distant metastases are treated with extended-field External Beam Radiation Therapy (EBRT) with concurrent platinum-based chemotherapy and brachytherapy [18]. Patients with a regionalized relapse of cervical cancer after the first treatment may be candidates for radical retreatment, including radiation therapy and/or chemotherapy and surgery [19]. The same treatment is recommended for LMICs. However, factors such as lack of infrastructure, adequate number of trained health care professionals, and expensive surgery affect effective treatment outcomes [19,20]. Like all other viruses, HPV depends on cellular machinery for replication [8]. Infection by HPV specifically subverts normal alternative splicing processes, producing isoforms impacting cellular functions and promoting carcinogenesis, cell proliferation, avoidance of immune response, and the inhibition of tumor suppressor proteins [8,21]. 

Epigenetic alterations, such as dysregulation of circular RNA (circRNA), long non-protein coding RNA (lncRNA), and microRNA (miRNA) levels, have been demonstrated to play essential functions in cell transmutation during several stages of cervical intraepithelial neoplasia and cervical cancer development [21,22]. Non-coding RNAs cross-regulate each other, forming the competing endogenous (ceRNA) networks [23]. ceRNA networks have, in recent years, been shown to play an essential regulatory function in promoting the incidence, development, and prognosis of cervical cancer [24]. Cervical cancer has ceRNA networks of circRNA, lncRNA, and miRNA that may be targeted to develop therapeutics or as biomarkers for screening [25,26]. For example, the combination of lncRNA MIR205HG and miRNA 122-5p fosters ceRNA-regulated cervical tumor cell proliferation and growth [27], and HOTAIR lncRNA by binding to miR-214-3p, may also act as a ceRNA, promoting cell proliferation and inhibiting cell death in HPV16^+^ cervical cancer cells [26]. Comprehensive studies on AS events and ceRNA networks’ regulation in cervical cancer are rare. This review will discuss ceRNA networks and splicing switches in cervical cancer and how these regulatory mechanisms are exploited by HPV in immune suppression and evasion in cervical cancer patients. Lastly, this review will deliberate on the therapeutic potential of splicing disruptor drugs. 

## 2. HPV Oncogenomics in Cervical Cancer

HPV is a small 7–8 kb, icosahedral, double-stranded, non-enveloped virus [28,29,30], with over 220 genotypes, which have been categorized into five genera, alpha, beta, gamma, mu, and nu [31]. Persistent HPV infections are the principal cause of cervical cancer. Other factors, including a weak immune system, multiple pregnancies, prolonged use of oral contraceptives, chlamydia infection, human immune deficiency virus (HIV), and smoking, can lead to cervical cancer [8,9,10,30,31]. HPV infections are classified as “High risk” (HPV 18 and 16) and “Low risk” (HPV 6 and 11) [10,30,32]. HPV16 causes 60% of cervical cancer, and HPV18 causes 15% [33]. High-risk HPV16 and 18 are responsible for about 70% of cervical cancer cases, HPV 31, 33, 45, 52, and 58~20% of the total cervical cancer cases [10,28,32,33,34,35]. The HPV genome is divided into three sections: a long non-coding area, an early region (E1, E2, E3, E4, E5, E6, E7, and E8 genes) encoding early oncogenic proteins, and a late portion (L1 and L2 genes) producing late proteins and viral encapsulation proteins. The E6 and E7 open reading frames encode proteins involved in cervical cancer development [36,37,38]. Like other genomes, little emphasis has been placed on non-coding genomic areas. 

**Figure 2:**
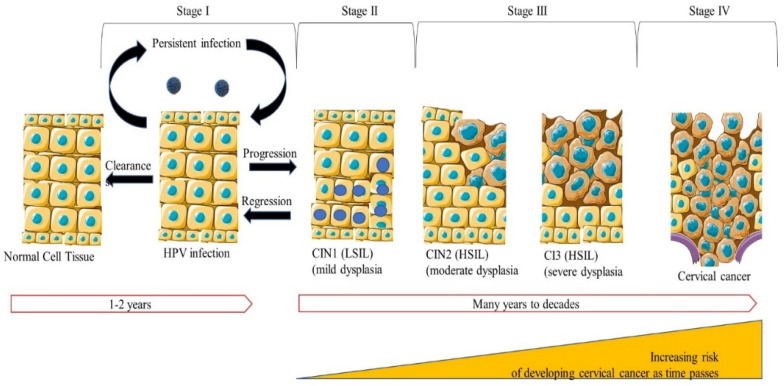
**HPV-mediated carcinogenesis in cervical cancer.** Infection with HPV mainly occurs in the cervical epithelium’s basal layer, which is exposed through abrasion. Infections are cleared by the immune system in 1–2 years; however, persistent infection results in the progression of infection. During the early stages of infection, early genes are expressed (E1, E2, E4, E5, E6, and E7), and viral replication occurs. The late genes encoding for late and viral encapsulated proteins are expressed, and the assembling of viral particles occurs. Lesions may progress to HSIL; however, this happens in a minority of women. If left untreated, HSIL usually leads to cervical cancer.

Carcinogenesis due to HPV can be divided into four stages in cervical cancer, as shown in Figure 2. Normal cells are infected with HPV, which in most cases is cleared within two years by the immune system. In 10% of the cases, the viral infection might persist, resulting in intraepithelial squamous lesions of low grade (LSIL)/CIN1, marking the second stage, distinguished by mild dysplasia. These lesions can progress to squamous intraepithelial lesions of high grade (HSIL)/CIN2 or in situ carcinomas (CIN3). HSIL can develop into invasive carcinomas, marking the fourth stage if left untreated [10,28]. Chances of spontaneous regression and clearance are common in CIN1 with a ~60% regression rate. High spontaneous CIN2 regression rates, 63%, and progression rates into CIN3 of 15% have been reported in young women aged 18–23. In women aged 18 to 62, 40% CIN2 regression was observed [39,40]. The risk of developing cervical cancer increases with time, 20% after five years and 50% after 30 years [8,28]. Cervical cancer, like other cancers, results from dysregulation in cell growth [30]. Constant genetic and epigenetic changes in HPV-infected cells allow for cervical cancer advancement. 

## 3. ceRNA Networks Regulate Alternative Splicing in Cervical Cancer

For decades, non-coding RNAs were neglected, and their operational roles were not thoroughly studied [41]. Non-coding RNAs are classified according to their sizes and functions, including small interfering RNAs (siRNAs), piwi-interacting RNAs (piRNAs), ribosomal RNAs (rRNAs), miRNAs (microRNAs), lncRNAs (long non-coding RNAs), small nuclear RNAs (snRNAs) and circular RNAs (circRNAs) [21]. 

Several RNA molecules, genes, and proteins interact with each other, forming a molecular network that promotes cervical cancer development and progression. Non-coding RNAs interact to create the ceRNA regulatory network [24]. The ceRNA network hypothesizes that RNA transcripts with miRNA response elements (MREs) can sequester from other targets, thus regulating their expression and cellular processes [42]. Recently, the ceRNA network has been shown to play a vital regulatory role in cervical cancer pathogenesis [24]. Evidence reveals that circRNA and lncRNA competitively bind to miRNAs and regulate downstream gene expression, forming the ceRNA regulatory axis [26,43]. The dysregulated expression of lncRNA, circRNA, and miRNA is involved in the processes leading to the development of cervical cancer, i.e., initiation and progression [22], as shown in Table 1. 

Based on current studies, ceRNAs play essential roles in alternative splicing regulation [21]. CeRNAs can control alternative splicing in a myriad of ways. One is by binding to cis-acting elements [62]. ncRNAs, acting as natural antisense transcripts (NATs), interact with pre-mRNA *cis*-acting elements via RNA-RNA base pairing. These interactions can influence splice site selection and splicing factor recruitment, consequently controlling alternatively spliced isoform expression in cancer [21]. Based on the origins of ncRNA, their interactions with *cis*-acting elements can be divided into *cis*-natural antisense transcript (NAT) and *trans*-NAT forms. *Cis*-NAT ncRNAs are transcribed from the complementary strand of DNA that codes for the target mRNA. Trans-NAT genes are transcribed from a divergent locus than the target mRNA [21,62]. Other mechanisms with which ceRNAs regulate alternative splicing are through regulating the mRNA expression of splicing factors (SFs), thus affecting the splicing isoform, controlling the posttranslational modifications of splicing factors (phosphorylation), rearranging chromatin, and influencing transcription via interacting with Polymerase ll and regulating DNA methylation [21]. 

**Figure 3:**
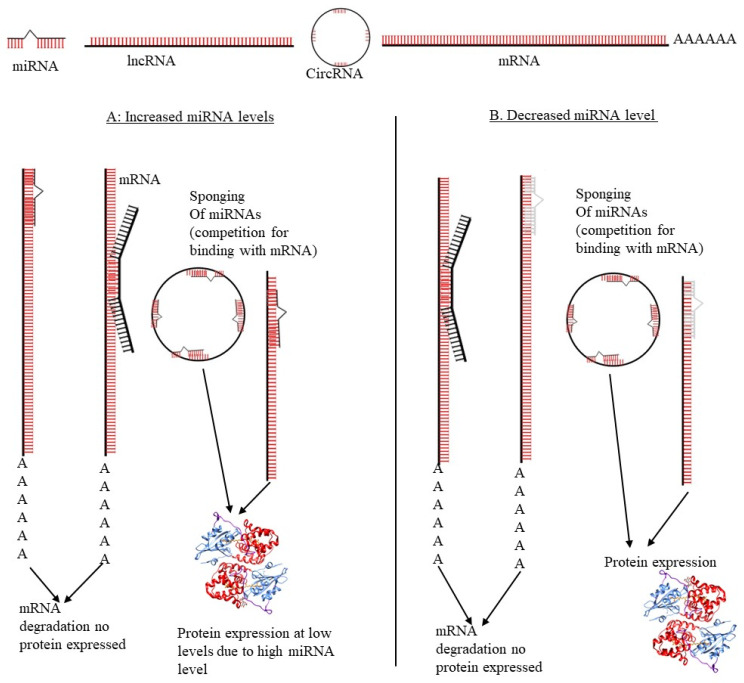
**ceRNA network regulation in cervical cancer.** mRNAs compete with lncRNAs and circRNAs for miRNA binding. (**A**) When upregulated, miRNAs bind to the 3’ UTR of mRNA and inhibit translation. However, this depends on the sponging ability of lncRNAs and circ-RNAs. (**B**) The down-regulation of miRNAs may promote protein translation from various transcripts (lncRNA, mRNA, and circRNA).

Emerging evidence has revealed the involvement of ceRNAs (miRNA-mRNA-lncRNA) in chemotherapeutic drug response in cervical cancer. Two possible mechanisms have been suggested; miRNA can act either as a promoter or an inhibitor of drug resistance. miRNA can bind to MRE in mRNA, thereby degrading or stopping mRNA’s translation, which may result in altered drug response due to the silencing of gene expression [62,63]. Inhibition of drug resistance occurs when increased levels of tumor suppressor miRNA expression downregulate or degrade the mRNA coding for an anti-oncogenic protein or a protein that inhibits drug resistance or increases sensitivity to the drug. This results in higher amounts of binding to the mRNA MRE and suppression of its translation. lncRNA and circRNA compete with mRNA for miRNA binding, preventing the degradation of drug resistance inhibitor mRNA [62]. These ceRNA networks may similarly promote or inhibit cervical tumorigenesis by inhibiting or upregulating oncogenic splice variants. As an oncogenic virus, HPV also encodes its own miRNAs that control the virus and host gene expression in favor of tumorigenesis, as shown in Figure 3. 

### 3.1. LncRNAs Role in ceRNA Networks Regulation and AS in Cervical Cancer

LncRNAs are regulatory transcripts longer than 200 nucleotides in length [9]. Numerous lncRNAs regulate the transcription of nearby genes, DNA repair, and the response to DNA damage, while some are involved in regulatory and structural functions, including splicing, epigenetics, signaling pathways as well as turnover and translation of mRNA [9,21,23]. lncRNA are thought to regulate downstream genes via competitively binding to miRNA at the post-transcriptional level; for instance, HOTAIR can control the miR-143-3p/BCL2 and miR-20a-5p/HMGA2 axis promoting cell growth and metastasis [26]. Li et al. [59] revealed an interaction between lncRNA SNHG4, c-Met, and miR-148a-3p, where SNHG4 upregulated c-Met through targeting miR-148a-3p and promoted cervical cancer development. In HPV 16 positive cervical cancer, Growth factor receptor-bound protein 2 (GRB2), responsible for cell communication, is highly expressed. MALAT1 is reported to indirectly influence GRB2 expression by interacting with miR-124 [55]. Several other ceRNA regulatory axes promote cervical cancer have been reported, as shown in Table 1.

LncRNAs play an essential role in regulating alternative splicing. LncRNAs influence alternative splicing by interacting with splicing factors. However, they can hijack splicing factors, causing dysregulation of alternative splicing. For example, Metastasis-associated lung adenocarcinoma transcript 1 (MALAT1), which is excessively expressed in cancer tissues and cervical cancer cells infected with “high risk” HPV [64], interacts with several serine-arginine proteins, namely SRSF1, SRSF2, SRSF3 and SRSF5 [65]. MALAT1 regulates serine-arginine proteins’ phosphorylation/dephosphorylation ratio, thus affecting their transportation and distribution to transcription sites and between nuclear speckle domains [45,65]. The mechanism for the phosphorylation of serine proteins by MALAT1 is still unclear; however, it might happen via interaction with PP1/2A phosphatases [66]. Studies demonstrate that MALAT1 can upregulate SRSF1-mediated splicing events that promote carcinogenesis (e.g., angiogenesis) [67]. In CaSki (HPV 16+) cervical cancer cells, MALAT1 encourages cell proliferation, migration, and cell cycle progression [68]. Other lncRNAs, such as MIR205HG, promote cervical cancer progression by targeting SRSF1 and regulating KRT17 [69].

LncRNAs can drive alternative splicing by interacting with cis-acting elements in pre-mRNA via RNA-RNA base pairing [21]. Interactions of the lncRNA (Saf) with cis-acting elements have been shown to promote exon skipping of Fas 6 through the recruitment of SPF45 to Fas pre-mRNA, causing the expression of soluble Fas. Soluble Fas promotes cancer progression by impeding Fas-FasL moderated apoptosis in numerous cancers, including cervical cancer [21,65].

### 3.2. miRNAs Role in ceRNA Network Regulation and AS in CC

miRNAs are a type of single-stranded RNA that is 20–22 nucleotides long and regulates gene expression by binding to sequence motifs found in the three ′ untranslated regions (UTR) of mRNA transcripts [22,23,70]. MiRNAs play a pivotal role in influencing gene expression and the development and growth of tumors [71]. Free circulating miRNAs are sponged by lncRNAs and circRNA, preventing them from interfering with transcription and obtaining the goal of gene expression [47]. Most often, miRNAs are tumour suppressers that target mRNAs that encode viral proteins and are involved in altered molecular functions in tumors [48]. MiR-124, a tumor suppressor, is reportedly low in cervical cancer due to being sponged up by MALAT1. The sponging up of miR-124 causes the upregulation of RBG2, resulting in cancer proliferation and invasion [55]. Many other miRNA including miR-1285-3p, miR-320a/, miR-337-3p, miR-8075, miR-136, miR-218, miR-1228-3p, miR-532-5p, miR-200a, miR-140-5p, miR-206, miR-143-3p, miR-148a, miR-383-5p, miR-143-3p, miR-127-5p, miR-3941, miR-148a-3p, miR-125b, miR-140-5p and microRNA-93 are sponged by lncRNA, and circRNA thus promoting cervical cancer development, invasion and metastasis, as shown in Table 1. Shang et al. 2022 [48] revealed downregulation of miR-532-5p, resulting in nodal metastasis. 

Studies have reported on the role of miRNAs in alternative splicing [21,62]. MiRNAs can regulate alternative splicing in multiple ways. Other miRNA functions as NATs, causing aberrant splice site selection in cancer. miRNAs also rearrange chromatin structure, affecting splicing factor recruitment, interact with Polymerase III, and influence histone modification and DNA methylation [62]. 

miRNAs are involved in regulating the expression of splicing factors. Splicing Factor expression can be inhibited by miRNAs through complete or partial complementarity to the target sequences of mRNA coding for SFs, leading to mRNA degeneration or translation downregulation [21]. Multiple miRNA-related influences on splicing factors have been discovered in many cancers, including cervical cancer. MiR-7, for example, inhibits SF SRSF1 mRNA translation in HeLa cells via partial complementarity with its 3’UTR, suppressing cancer cell survival. MiR-221, miR-222, and miR-17-92 are also known to influence SRSF1. SRSF1 is involved in the expression of various cancer-promoting genes, including pro-apoptotic Bcl-x, R.O.N., and MCL-1 isoforms [62] and activating SRSF1 through its kinase SRPK1 is activated by HPCV infection [72]. MiR-802 is shown to target SRSF9 and cause apoptosis in cervical cancer [73]. CircRNAs and lncRNAs have increased SF expression in cancers, including cervical cancer, by regulating alternative splicing [21].

### 3.3. circRNAs Role in ceRNA Network Regulation and AS in Cervical Cancer

There is a growing concern among researchers on the role played by circRNA in cancer, including cervical cancer [74]. Studies have implicated circRNAs in cervical cancer development, aggression and progression not only through deregulating chromatin modifications but additionally through competitively binding to miRNA to regulate the expression of genes [44]. CircRNA such as circRNA_400029 and circEPSTI1 promote cancer growth, invasion and inhibits cell death via regulating the miR-1285-3p/TLN1 and miR-375/409-3P/515-5p-SLC7A11 axis, respectively [44,74]. CircSLC26A4 encourages cervical cancer growth by regulating the miR-1287-5p/HOXA7 axis [74]. These studies suggest that circRNAs regulate cancer development and progression through downregulating miRNAs. Song et al. 2020 [75], revealed that circRNA-101996 downregulates miR-8075 and upregulates TPX2 expression. Furthermore, it was suggested that a single circRNA could sponge several miRNAs. For example, circRNA_101996 could sponge miR-8075 and miR-1236-3p. Various other circRNA have been shown to play a regulatory role in cervical cancer, as shown in Table 1.

The functions of circRNA are unknown; however, studies have shown that they are generated through back splicing of mRNA [76]. CircRNAs play a critical role in regulating alternative splicing. Furthermore, circRNA can enlist or inhibit particular proteins from acting as scaffolds to aid protein-enzyme reactions. circRNA have been observed to encourage splicing Factor expression by sponging miRNAs, thus regulating alternative splicing in malignancies [21]. Moreover, circRNA are involved in RNA splicing and mRNA by acting as a sponge for ribonucleoprotein [77]. It has been observed in glioblastoma multiforme that circRNAs like circRNA cir-c SMARCA5 are able to regulate the expression of VEGF-Axxxa pre-mRNA through binding to the SF SRSF1 [21]. However, there is limited literature on the involvement of circRNAs in alternative splicing in cervical cancer.

PRMTs are overexpressed in several cancers, including cervical cancer [50,78,79]. Alternative splicing of PRMT genes produces novel circRNAs implicated in several cancers, for example, splicing of the PRMT1 gene in breast cancer [80] and circPRMT5 in non-small cell lung cancer and bladder cancer [81]. Functional cooperation exists between PRMTs and ncRNAs resulting in a net upregulation of PRMTs in cancers [82].

Small RNA expression profiling in cervical neoplasia revealed upregulated “oncogenic” miRNAs like miR-19, miR-146a, miR-21, and miR-10a, as well as down-regulated “tumor-suppressive” miRNAs like miR-29a, miR-218, miR-214, and miR-372, which are involved in cell proliferation, neoplastic transformation, cell migration, and invasion [22]. The HPV genome encodes the HPV-16-miR-H1-1 and HPV-16-miR-H2-1, capable of targeting essential cell genes such as those governing cell cycle progression, migration, and immune response, in addition to being required for viral infection and upkeep [83].

LncRNAs can bind mRNAs, proteins, or miRNAs and are entangled in several biological functions. The deregulation of lncRNA expression has been linked to cardiovascular and neurodegenerative diseases and cancer development. Multiple lncRNAs such as HOTAIR, H19, MALAT1, CCAT2, SPRY4-IT1, GAS5, CCHE1, MEG3, LET, EBIC and PVT1 are thought to play essential roles in cervical cancer growth, invasion and metastasis, and radio-resistance [22]. CircRNAs, like lncRNA, act as a sponge for miRNA competing for mRNA binding. Circ-0018289 has been shown to sponge miR-497 and contribute to the dysregulation of target genes [22,84]. Viral agents encode for circE7, which overexpresses the E7 oncoprotein, thus driving cell transformation [22]. The generation of lncRNAs is part of the process of alternative splicing of the human genome [65]. For instance, in lung adenocarcinoma, breast cancer, and colorectal adenocarcinoma, it has been observed that the splicing factor hnRNPE1 binds to the PNUTS 5′ pre-mRNA exon 12 splicing site, promoting the production of PNUT mRNA. The dissociation of hnRNPE1 induces the formation of lncRNA PNUTs isoforms, which are implicated in epithelial-to-mesenchymal transition (EMT), resulting in tumor progression. The lncRNA PNUTS acts as competitive sponges for miR-205, inhibiting miR-205 binding to the ZEB1 gene, thus causing an upregulation of ZEB1. The upregulation of ZEB1 inhibits the expression of E-cadherin, thereby inducing Epithelial-mesenchymal transitions (EMT) and tumor progression [65,85]. Despite the fact that Farzanehpour et al. [86] revealed an upregulation of Snail1 and ZEB1 levels and reduced expression levels of E-cadherin in cervical cancer samples, a link between this upregulation and the ceRNA has not been reported

## 4. Role of AS and ceRNA Networks in HPV Immune Suppression and Evasion in Cervical Cancer

During infection by a virus, modulation of alternative splicing could be activated by cells as a defense strategy to impede the virus or caused by viral proteins and RNA [87]. Furthermore, tumors can avoid detection by the immune system through the alternative splicing of apoptosis-related genes, particularly the *Bcl2* family and many caspases, forming transcripts supportive of cancer development or progression [88]. Even though reported by several studies, the exact mechanisms by which oncoviruses, especially HPV in cervical cancer, exploit the host splicing machinery remain largely to be elucidated. RNA molecules forming the ceRNA network have gradually been demonstrated to play an essential function in the tumor immunosuppressive environment (TIE) [89]. In the last few years, several studies have revealed that ncRNAs can directly or indirectly affect the TIE in cervical cancer. For example, lncRNA 00518 and lncRNA SNHG14 are shown to activate the JAK2/STAT3 signaling pathway [89,90] and lncRNA UICC upregulates IL-6 and activates the STAT3 signaling pathway inducing immunosuppression [91]. 

It has also been reported that ncRNAs can target elements of the TGFβ- signaling pathway or directly regulate TGFβ-target gene transcription in various tumors, thereby impeding antitumor immune responses [89]. In addition, miR-21 regulates Smad7 expression, decreasing sensitivity to chemotherapy in cervical cancer [92]. However, the dysregulation of miR-21 promotes immune escape [92]. Additionally, LOC105374902 lncRNA was found to promote the cancerous behavior of cervical cancer cells by sponging up for miR-1285-3p, which is behind the inhibition of cell proliferation, invasion and migration, when induced by TNF-α. This multifunctional cytokine can regulate inflammation and immunity in cancer [93,94]. 

A recent study demonstrates that tumor cells may increase the expression of HLA molecules, including HLA-G, to escape immunosurveillance. HLA-G binding to inhibitory receptors on varied immune cells leads to inhibitory immune responses, like the down-regulation of CD8+ T cell and NK cell cytotoxicity [95]. HOTAIR, a lncRNA, could significantly alter the expression of HLA-G in cancer cells by binding to miR-152 or miR-148a competitively. The increased HOTAIR expression was connected with more enhanced clinical features and substantially shorter survival in patients with cervical cancer [96]. Although ceRNA networks are involved in tumor immune escape, immune-related ceRNA networks and splicing switches are rarely reported in cervical cancer.

## 5. Clinical Significance: ceRNA Networks Regulation and mRNA Splicing Switches in Cervical Cancer

Mainstream cancer treatments are only efficacious in a subset of patients with an increased chance of resistance [97]. Cancer transcriptomes differ significantly from normal cells, as do ceRNA profiles in malignancies [98]. Studies show that ceRNA expression is deregulated in cancers [99,100], and ceRNAs are comparably stable and can be detected in bodily fluids [101]. The ceRNA network has proven to be an efficient means of identifying potential diagnostic and prognostic biomarkers in several cancers [70,102]. The discovery of efficient drug-specific targets that target cancer cells without harming healthy cells will thus aid in improving the clinical outcome of cervical cancer.

Approximately 46% and 10% of cervical cancer patients are diagnosed with early-stage, and late-stage disease, respectively, and cervical cancer incidences are increasing [103]. It is therefore imperative to identify biomarkers specific and sensitive to cervical cancer. Through bioinformatic analysis, Li et al. showed that 129 lncRNAs, 8 miRNAs, and 298 mRNAs are linked with cervical squamous cell carcinoma and endocervical adenocarcinoma prognosis [101]. lncRNA GIHCG, highly expressed in cervical cancer, can distinguish cervical cancer patients from healthy controls with a sensitivity and specificity of 88.75 and 87.50%, respectively, making it a possible diagnostic biomarker [104]. Another probable diagnostic lncRNA is lnc-PVT1 [105], transcribed from the 8q24 chromosome [106]. Due to its high serum expression in cervical cancer, it predicts and gives the prognosis of the disease, making it an accurate diagnostic biomarker [105]. 

Other lncRNA with diagnostic potential include AC126474 and C5orf66-AS1, which predict cervical cancer metastasis. LncRNAs GHET1 and SOX21-AS1, both linked to lymph node metastasis, poor histological grade, and advanced clinical stage, can be used to estimate cervical cancer survival rate [103]. HOTAIR, GAS5, TUSC8, and lncRNA-LET may be valuable biomarkers for predicting cervical cancer prognosis, as they are associated with invasion and metastasis [107,108]. The inhibition of miR-143-5p by lncRNA-TCONS 00026907 acts as a prognostic indicator in cervical cancer [69]. Aberrantly expressed circRNAs are abundant in cervical cancer and promote cancer progression [109]. CircRNAs circulate freely in human saliva, urine, and blood, elevating their potential as non-invasive biomarkers with diagnostic value [110]. Wang et al. detected 80,000 circRNAs in cervical tumors and healthy tissue, with 25,000 circRNA expressed differently [45]. Upregulated hsa_circ_0018289 and circ_0067934 were revealed to favour neoplastic growth in vivo. The elevated expression of circRNA8924 and circ_0067934 corresponded with myometrial invasion and poor overall survival, respectively, serving as biomarkers for cervical cancer [109,111]. Based on the Kaplan–Meier curve analysis, hsa_circ_0023404 can act as a prognostic marker as its overexpression in patients with cervical cancer foretold poor prognosis [112]. Jiang et al. showed that miRNAs could be biomarkers in diagnosing CIN and cervical cancer [113]. Moreover, miRNAs distinguish between CIN patients and healthy individuals with great diagnostic performance [113]. Since miRNAs are stable in the circulating system and are shown to possess greater efficacy than cervical tissues due to the invasiveness when harvesting samples. The upregulation of serum miR-−25, −200a, −21, −29a, and −486-5p presents the miRNAs as non-invasive, accurate biomarkers for cervical cancer [114].

According to growing evidence, alternative splicing isoforms have surfaced as valuable biomarkers in carcinomas’ development, growth, and outcome, potentially providing new treatment targets for malignant tumors, including cervical cancer [115,116]. Five genes (HNRNAP1, PLAU, HNRNPAB, FES, and POMGNT1) with aberrant splicing events were prognostic in cervical cancer [117]. Furthermore, aberrant alternative splicing of PRMT1 has been reported in various cancers, including cervical cancer [118,119]. PRMT splicing switches are emerging as essential role players in tumorigenesis, with PRMT5, 6, and 8 overexpressed in cervical cancer [120]. The elevated expression of PRMT5 suggests that PRMT5 may be a possible biomarker for numerous cancers, including cervical cancer [121]. PRMT regulation occurs in either the nucleus or the cytoplasm., making it arduous to use PRMT expression as biomarkers before acquiring tissue samples [78]. Further research is required to determine whether other PRMTs can be used in the diagnosis and prognosis of cervical cancer.

## 6. Epigenomics of PRMTs and Alternative Splicing

PRMTs are reportedly overexpressed in several cancers, including cervical cancer, and play a vital role in controlling splicing switches [78,122]. PRMTs are enzymes behind the posttranslational modification of proteins. i.e., Arginine methylation [119]. The human genome encodes for nine PRMTs, PRMT1 to PRMT9 [123,124], further categorized into three types based on the final methyl-arginine residue [118,125]. Type I includes PRMT 1,2,3,4,6 and 8, responsible for the generation of ω-N^G^-mono- methyl-arginine (MMA) and ω-N^G^, N^G^-asymmetric dimethylarginine (ADMA); type II includes PRMT5 and 9, involved in catalyzing the generation of ω-N^G^-mono- methyl-arginine (MMA) and ω-NG, NG-symmetric dimethylarginine (SDMA); and type III has PRMT7 responsible for catalyzing the formation of ω-N^G^-mono- methyl-arginine [125,126]. The most common is PRMT1, accounting for >90% of the methylated proteins in mammalian cells [127].

Due to the significance of arginine methylation to protein functionality, PRMTs are revealed as participants in transcriptional and post-transcriptional regulation, cell cycle checkpoints, phase separation, DNA damage repair [123,125], mRNA metabolism, and intracellular signaling during cancer development and progression [126]. Notably, the methylation of several protein substrates catalyzed by PRMTs is involved in numerous cancer processes, including initiation, progression, and metastasis [82]. Evidence also suggests that arginine methylation drives the aggressiveness of cancer [118]. However, each enzyme’s specific roles in carcinogenesis are not understood [3]. Alterations in PRMTs are rare; however, upregulation of PRMTs has been linked to cancer, making them targets for therapeutic intervention [127]. 

Multiple studies show that alternative PRMT isoforms are upregulated in several cancers (non-small cell lung, cervical, prostate, and breast cancer) [118,128,129]. Studies revealed that PRMT5, PRMT6, and PRMT8 mRNA and proteins are overexpressed in cervical cancer and are linked to poor survival in cancer patients [78,120]. However, the mechanism with which they aid cancer progression is poorly studied [120]. Inhibition of PRMTs causes changes in alternative splicing [126] and reduces protein translation [125]. The precise role of HPV-mediated tumorigenesis in cervical cancer by the overexpression of PMRTs remains to be elucidated. Still, the fact that arginine methyl transferase inhibitor acts by reducing PRMT5 to inhibit cervical cancer growth suggests that specific mechanisms exist [79]. PRMTs are concerned with the splicing of genes responsible for cell survival, differentiation, and proliferation. PRMTs are revealed to influence alternative splicing in a methylation-dependent manner [130]. The functions of PRMT2-4, 6, and 8 in splicing remain unclear [124]. 

**Figure 4:**
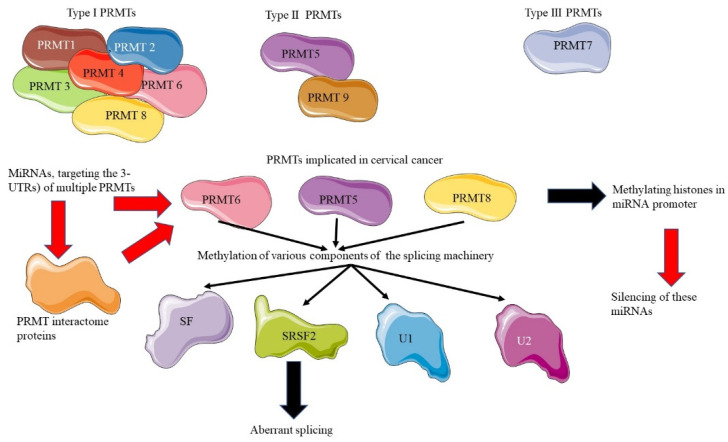
**PMRT enzymes and alternative splicing.** PRMTs regulate alternative splicing through the methylation of multiple components of the splicing machinery. The aberrant expression of PRMTs results in aberrant splicing, driving the development of cervical cancer. PRMT5, 6 and 8 are reportedly overexpressed in cervical cancer. A cross-regulatory relationship between miRNAs and PRMTs promotes cancer development and progression. miRNAs target the 3-UTRs of PRMTs for degradation, and PRMTs methylate histones in the miRNA promoter, leading to the silencing of miRNAs.

However, other PRMTs are shown to regulate elements involved in RNA splicing, as shown in Figure 4. PRMT1 regulates the function and localization of RNA binding proteins (RBPs). PRMT5 is responsible for maturing small nuclear ribonucleoproteins and upholding splicing precision. Maturation of snRNPs is facilitated by the symmetrical di-methylation of RG/RGG motifs in Sm proteins (snRNBP, snRPB, snRPD1, snRPD3), which leads to their identification by the Tudor domain of the survival motor neuron (SMN). The Tudor domain binds the Sm proteins and snRNA, aggregating them into fully developed snRNPs [127]. 

In the cytoplasm, the spliceosome-associated protein SF3B2 (also named protein 145) is monomethylated and di-methylated by PRMT9, facilitating its binding to the S.M.N. Tudor domain, thus regulating alternative splicing [126,131,132]. In the nucleus, PRMT4 (CARM1) controls AS via the methylation of splicing factors, co-regulators, RNA polymerase II, and transcription factors and promotes exon skipping [127,131]. CARM1 methylates various proteins associated with pre-mRNA processing and enables splicing reporter and endogenous CD44 gene skipping [130]. CARM1 asymmetrically di-methylates splicing factors, namely SAP49, U1 snRNP, and transcription factor CA150. CARM1, like PRMT5, can symmetrically methylate Sm proteins [131]. CARM1 promotes the proliferation of MFC-7 breast cancer cells [127]. The role of PRMTs in alternative splicing is a relatively new field of research; therefore, the role of PRMTs in promoting cervical cancer pathogenesis is understudied. There is, therefore, no conclusive proof that PRMTs are involved in the alternative splicing of cervical cancer. 

While ceRNA network regulation can hinder or promote aberrant splicing switches that may promote tumorigenesis, there is a lack of reports in this area. PRMTs may potentially impede the tumorigenic effects of ceRNAs networks by disrupting the oncogenic splicing switches. The virus and the host produce distinct mRNA transcripts and ceRNA networks. However, how these RNA molecules interact with PMRTs in cervical cancer remains mysterious. 

Furthermore, studies have recently shown that there is a mutual regulation between miRNAs and PRMTs which results in harmonized control of many facets of cancer progression, such as altered gene expression, cell migration, invasion, and cell stemness maintenance. PRMTs regulate miRNAs by methylating histones in miRNA promoter regions, such as H3R8me2s, H4R3me2s, and others, resulting in the silencing of these miRNAs’ transcription. MiRNAs, on the other hand, regulate PRMTs at various levels, such as targeting the 3′-untranslated regions (3-UTRs) of multiple PRMTs for degradation or obliquely via silencing of PRMT interactome proteins. This co-regulation results in the upregulation of PRMTs that encourage cancer development [82]. Although the mutual regulatory effect of ceRNA and PRMTs have been reported in several cancers, as discussed below, their impact on cervical cancer is yet to be studied. CircRNAs such as circ-PRMT5 (hsa_circ_0031242) produced by the PRMT5 gene promote bladder cancer development by regulating EMT through sponging up miR-30c [133]. The tumor-promoting effect of circ-PRMT5 was also established in lung cancer [134] and gastric cancer via sponging up miR-145 and miR-1304 [135]. In non-small-cell lung cancer (NSCLC), an upregulation of circ-PRMT5 and a down-regulation of miR-4458 suggests that circ-PRMT5 is a ceRNA for miR-4458. This inverse regulation confers chemoresistance to NSCLC [136]. Ma et al. [137] demonstrated that in breast cancer, circ-0039960 positively mediates PRMT7 expression through targeting miR-1178, thus regulating cell growth and the Warburg effect. The regulatory impact of PRMTs and the ceRNA network in cervical cancer has not been elucidated. However, since PRMT5 is upregulated in cervical cancer [120], this could suggest overexpression of circ-PRMT5, which possibly drives cervical cancer progression by sponging miRNAs such as miR-145. Even though there are currently limited studies to fully demonstrate and comprehend PRMTs’ role in cervical cancer aberrant splicing switches, this review highlights a research gap that needs to be filled in comprehending this phenomenon.

## 7. PRMT Inhibitor Drugs’ Therapeutic Potential

The implication of PRMT dysregulation in cancer development and progression has prompted their use as therapeutic targets in anticancer drug development [122,126]. Consequently, the focus has been shifted to developing effective and selective PRMT inhibitors because each isoform has its distinct function [3,122]. In recent years, there has been growing curiosity about the mechanisms that support using PRMTs in cancer therapy [138]. Several small molecule inhibitors targeting PRMTs have been identified, and several are in the pre-clinical trial stage. Inhibitors developed to date are highly effective against type I compared to type II PRMTs [132], particularly CRAM1, PRMT1, and PRMT5.

Mutations in RNA splicing factors often cause dysregulation in alternative splicing, and inhibition of type I PRMT1 and PRMT5 by splicing disruptor drugs is shown to occur in cells with RNA splicing factor mutations, both in vitro and in vivo [138]. The first type I PRMT inhibitor to be discovered was AMI-1, revealed to inhibit solid tumors in cervical cancer by targeting PRMT 5. The discovery of AMI-1 prompted the need for more specific inhibitors, leading to the developing of more potent drugs, including GSK3368715 and MS023. MS023 inhibits methylation of ADMA and increases MMA and SDMA methylation in mantle cell lymphoma [131]. Inhibition of PRMT1 affects the methylation of RNA binding proteins and the assembling of spliceosomes, making it a therapeutically effective strategy [132]. Treating pancreatic cells with PRMT inhibitors is shown to cause significant aberrations in splicing, with exon skipping as the most frequent alteration affecting essential pathways such as the cell cycle and mitosis [139,140].

**Figure 5:**
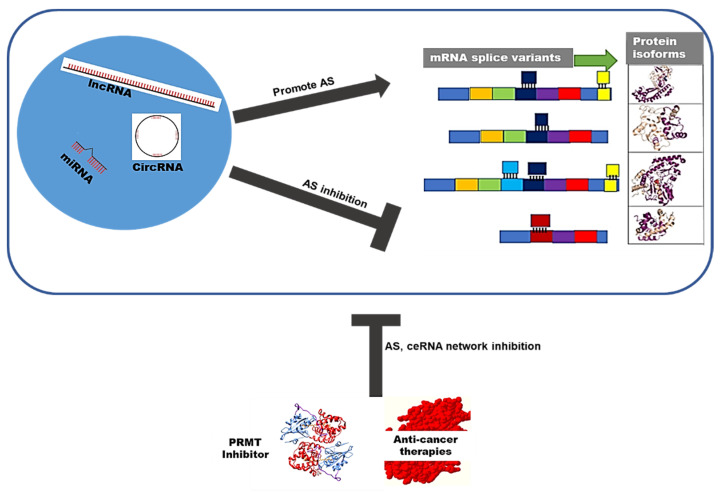
**PMRT inhibitors drug therapeutic potential.** The ceRNA network regulates AS by encouraging or inhibiting the generation of protein isoforms. PRMT inhibitor drugs can counter the effects of the ceRNAs network by disrupting AS. PRMT inhibitors may also be used with other anticancer therapies to enhance their efficacies.

Several studies have shown the antitumor activity of PF-0639999, a type II inhibitor, rationalizing its use in treating splicing dysregulation in lung cancer [141]. Combination treatment has been revealed to be more effective in killing tumor cells. In pancreatic cancer cells, the combination of GSK3203591 and GSK3368715 is more effective as increased concentrations of each drug enhance the potency of the other. Moreover, cytotoxicity against the cell lines increased at concentrations where each drug was cytostatic [140]. PMRT5 inhibitors can be synergized with other anticancer therapies; for example, GSK3326595 can assist palbociclib (CDK4/6 inhibitor) in overcoming resistance in patients with melanoma [142]. Although PRMTs are overexpressed in cervical cancer [120], limited literature examining the sensitivity of cervical cancer to PRMT inhibitors exists. Including PRMT inhibitors in clinical trials opens an exciting research area in the arginine methylation field. Understanding the downstream pathways regulated by PRMTs will open opportunities to exploit vulnerabilities in cancer cells, especially in cancers with a viral etiology such as cervical cancer [118]. Figure 5 demonstrates the therapeutic potential of PRMT inhibitor drugs, used to target alternative splicing (AS) and can also be used to enhance the efficacy of other anticancer therapeutics. 

## 8. Limitations and Challenges

Despite cervical cancer being the fourth most prevalent cancer globally and its causative agent, HPV, using alternative splicing to advance latency, limited research exists examining how alternative splicing aids immunosuppression. The literature does not clearly define the connection between ceRNAs and alternative splicing in cervical cancer. The implication of PRMT dysregulation in cancer development and progression has prompted their potential use as therapeutic targets in anticancer drug development. Several small inhibitor molecules targeting PRMTs have been used in clinical trial phases of breast, colon, pancreatic, and kidney cancer. However, despite PRMTs being reportedly overexpressed in cervical cancer, limited research studies have reported the use of PRMT inhibitors in cervical cancer. Improving cervical cancer research can increase the understanding of how HPV drives pathogenesis, especially in cohorts with HIV and identify potential biomarkers which would be beneficial for LMICs, where HIV infection and cervical cancer are colliding with public health burdens. While PMRT small molecule inhibitors are emerging as splicing disruptor drugs in solid tumors, there is still a lack of evidence to define the precise roles of these drugs in viral-induced cancers, especially in HPV viral oncogenomics in cervical cancer. HPV encodes for few proteins; however, its immune system evasion ability through exploiting the host splicing machinery and lack of HPV-targeted small molecule inhibitors warrants innovative approaches. 

## 9. Conclusions

Cervical cancer is a challenge in LMICs. While HPV is the number one cause of cervical malignancy, other factors, including smoking, oral contraceptives, and HIV, contribute to the progression of the disease. HPV is reported to advance latency through alternative viral and host mRNA splicing. This article discussed the role of HPV immunosuppressive effects in cervical cancer by exploiting the splicing machinery. HPV dysregulates alternative splicing resulting in the formation of isoforms that help the virus evade detection by the immune system. The role of alternative splicing in immunosuppression is an understudied area and closing this gap in the research would aid in understanding how viruses utilize this process. CeRNAs regulate splicing through binding to cis-acting elements and regulating the expression of splicing factors. Dysregulation in the expression of Splicing Factors promotes cancer cell survival. Additionally, ceRNAs networks’ regulation in HPV-mediated immunosuppression in cervical cancer is underreported, and this RNA cohort can be further studied to fully comprehend HPV pathogenesis in cervical cancer. Like ceRNAs, PRMTs control components involved in splicing and are upregulated in cervical cancer. The role of most PRMTs in mRNA splicing remains to be elucidated. However, emerging reports show they can be potential targets for therapeutic interventions. 

Although PMRT inhibitors have been developed, more potent and specific PRMT inhibitors are needed. These will target each specific PRMT, thereby increasing their effectiveness. This review discussed the underreported research areas of the role played by AS in immunosuppression in cervical cancer, regulatory roles of AS and ceRNA networks in HPV-induced cervical cancer pathogenesis, PMRT roles in cervical cancer, and the therapeutic potential of inhibitors of PMRTs, which may be used cancers with a viral etiology such as cervical cancer. The HPV–host transcriptome landscape is broad, resulting in a knowledge gap that warrants further elucidation to favor cervical cancer patients’ outcomes.

## Figures and Tables

**Table 1:** CeRNA regulatory axis in cervical cancer.

ceRNA	Regulatory Axis	Role in Cervical Cancer	Ref
circRNA 400029	miR-1285-3p/TLN1	Aggressive behaviors of cervical cancer	[44]
circCLK3	MiR-320a/Fox M1	Cervical cancer progression	[43]
hsa_circ_0001038	miR-337-3p/cyclin-M3	Promotes cell growth, migration, and invasion	[45]
hsa_circRNA_101996	miR-8075/TPX2	Promotes cell growth and invasion	[45]
hsa_circ_0023404	miR-136/TFCP2	Cervical cancer development and progression	[25]
circ-EIF4G2	miR-218/HOXA1	Modulates malignant biological behaviors	[46]
Hsa_circ_0000301	miR-1228-3p/IRF4	Cancer progression	[47]
miR-532-5p	LINC01410/FASN	Tumour metastasis	[48]
LncRNA XIST	miR-200a/Fus	Cancer progression	[43]
LncRNA XIST	miR-140-5p/ORC1	Cell proliferation and increased expression of Bcl-2	[49]
LncRNA HOTAIR	miR-206/MKL1	Migration and invasion	[50]
LncRNA HOTAIR	miR-143-3p/BCL2	Inhibit tumor suppression	[51]
LncRNA HOTAIR	miR-148a/human leucocyteantigen-G (HLA-G)	Proliferation, migration, and invasion of cervical cancer cells	[52]
LncRNA NEAT1	miR-133a/Sox4	Cell proliferation, migration, and invasion	[53]
LncRNA LINC01128	miR-383-5p/SFN	Inhibits apoptosisproliferation, migration, and invasion of cervical cancer cells	[54]
LncRNA MALAT1	miR-124/RBG2	Proliferation, migration, and invasion	[55]
lncRNA OIP5-AS1	miR-143-3p/ROCK1	Inhibit apoptosis and promotes cell proliferation	[56]
LncRNA RNA POU3F3	miR-127-5p/FOXD1	Promoted the proliferation and invasion	[57]
LncRNA RP11-552M11.4	miR-3941/ATF1	Cell proliferation	[58]
SNHG4	miR-148a-3p/c-Met	Improve cell viability and inhibit apoptosis	[59]
SNHG12	miR-125b/STAT3	Proliferation and invasion of cervical cancer	[59]
lncRNA SU1P2	let-7a/IGF1R, let-7a/N-myc, and let-7a/EphA4	Promotes tumorigenesis	[55]
LncRNA SNHG20	miR-140-5p-ADAM10	Promote cervical cancer cells proliferation and invasion	[60]
ZNF667-AS1	microRNA-93-3p/PEG3	Decreases tumor invasion and metastasis	[61]

## Data Availability

Not applicable.
